# Determinants of psychotherapists’ attitudes to online psychotherapy

**DOI:** 10.3389/fpsyt.2023.1196907

**Published:** 2023-06-22

**Authors:** Emilia Rutkowska, Joanna Furmańska, Hakan Lane, Cristiana C. Marques, Maria João Martins, Najam us Sahar, Johannes Meixner, Valeria Tullio, Antonina Argo, David Marcelo Bermeo Barros

**Affiliations:** ^1^Institute of Psychology, University of Szczecin, Szczecin, Poland; ^2^Brandenburg Medical School Theodor Fontane, Brandenburg an der Havel, Brandenburg, Germany; ^3^Center for Research in Neuropsychology and Cognitive and Behavioral Intervention (CINEICC), Faculty of Psychology and Educational Sciences, University of Coimbra, Coimbra, Portugal; ^4^University of Coimbra Health Services, University of Coimbra, Coimbra, Portugal; ^5^Fatima Jinnah Women University, Rawalpindi, Punjab, Pakistan; ^6^Department of Health Promotion, Maternal and Child Care, “G. D’Alessandro”, University of Palermo, Palermo, Sicily, Italy; ^7^Department of Health Promotion, Maternal and Child Care, “G. D’Alessandro”, University of Palermo, Palermo, Italy, University of Palermo, Palermo, Sicily, Italy; ^8^Neuroscience Group, University of Azuay, Cuenca, Azuay, Ecuador

**Keywords:** online therapy, therapist factor, psychotherapeutic practice, attitudes, COVID-19, international research, online psychotherapy

## Abstract

**Introduction:**

Online psychotherapy is a form of work that is becoming more and more popular. Public health problems, such as COVID-19, forced mental health professionals and patients to incorporate new methodologies such as the use of electronic media and internet to provide follow-up, treatment and also supervision. The aim of this study was to investigate which factors shape the therapists’ attitudes toward online psychotherapy during a pandemic taking into account: (1) attitudes toward the COVID-19 pandemic (fear of contagion, pandemic fatigue, etc.), (2) personal characteristics of the psychotherapists (age, gender, feeling of efficacy, anxiety, depression, etc.), and (3) characteristics of the psychotherapeutic practice (guideline procedure, client age group, professional experience, etc).

**Materials and methods:**

Study participants were 177 psychotherapists from four European countries: Poland (*n* = 48), Germany (*n* = 44), Sweden (*n* = 49), and Portugal (*n* = 36). Data were collected by means of an individual online survey through the original questionnaire and the standardized scales: a modified version of the Attitudes toward Psychological Online Interventions Scale (APOI), Fear of Contagion by COVID-19 Scale (FCS COVID-19), Pandemic Fatigue Scale (PFS), Hospital Anxiety and Depression Scale (HADS), Social Support Questionnaire (F-SozU K-14), and the Sense of Efficiency Test (SET).

**Results:**

Determinants that impacted psychotherapists’ attitudes toward online therapy were: COVID-19 belief in prevention—keeping distance and hand disinfection, pandemic behavioral fatigue, previous online therapy experience (including voice call), working with youth and adults. Our study showed that belief in the sense of prevention in the form of taking care of hand disinfection before the session, pandemic behavioral fatigue and experience in working with adults were significant predictors of negative attitudes of therapists toward online psychological interventions. On the other hand, belief in the sense of prevention in the form of keeping distance during the session had a positive effect on general attitudes toward therapy conducted via the internet.

**Discussion:**

The online therapy boom during the COVID-19 pandemic has spawned a powerful tool for psychotherapists. More research in this area and training of psychotherapists are needed for online psychological interventions to become an effective therapy format that is accepted by patients and therapists alike.

## 1. Introduction

Over the past years, changes in counseling service provision have been observed such as the influx of telemedicine and online counseling sessions (1). In particular, the situation during the COVID-19 pandemic prompted therapists and patients to seek help through technology. Research shows that online counseling has both positive and negative effects in terms of accessibility, effectiveness, ethical considerations, and client-therapist relationship (2). More recently, the positive effects of working online have been confirmed by a vast number of studies (3–8). In comparing the difference between online and face-to-face counseling, Murphy et al. (9), concluded that client satisfaction levels were similar in both conditions. Despite the available evidence confirming the overall effectiveness of online therapy, therapists still keep reservations about this setting (10, 11). Therapists reported that they feel more tired, less competent, less confident, and less authentic among other negative aspects and difficulties of online work. Moreover, they felt less connected to the clients during the online sessions. Together, these negative feelings were associated with more negative attitudes toward online psychotherapy (1, 2).

In the present study, we were particularly interested in what factors influence the therapists’ attitudes toward online psychotherapy. In our analysis, we considered three groups of factors: (1) Feelings and thoughts related to the COVID-19 pandemic (fear of becoming infected with COVID-19, pandemic fatigue, commitment to infection prevention, etc.); (2) personal characteristics of the psychotherapists (age, gender, sense of efficacy, social support, anxiety, depression, etc.); and (3) characteristics of the psychotherapeutic practice (therapeutic approach, client age group, setting of psychotherapy, years of professional experience, experience with online therapy, etc.). The primary research question of the current study therefore aims at the interaction of these three groups of factors (attitude toward the pandemic, constitution of the psychotherapist, and psychotherapeutic approach) as well as their associative relation with psychotherapists’ attitudes toward online psychotherapy. Which factors are crucial for the way how psychotherapists conduct and evaluate online psychotherapy in times of the COVID-19 pandemic?

## 2. Materials and methods

### 2.1. Procedure, participants, and recruitment

An anonymous longitudinal online survey was conducted in four European countries, namely Poland (Eastern Europe), Germany (Western Europe), Sweden (Northern Europe), and Portugal (Southern Europe). Individuals working as professional psychotherapists were recruited through advertisements on social media platforms or professional associations, direct enquiries to publicly available email addresses, on the websites of professional associations or therapy practices, or through professionals of the author’s circle. A reminder was sent up to two times. After obtaining informed consent from the study participants, data were collected via an online survey in their native language. Participants did not receive any financial compensation. Data collection started in February 2022 and ended in March 2022.

### 2.2. Measures

#### 2.2.1. Original questionnaire

We specifically designed a questionnaire to collect information about a professional therapist’s working style and variables related to experiences in conducting online therapy, as well as variables related to working in the context of the risk of contracting COVID-19. For the present study, we collected data related to the profession of a psychotherapist practice, e.g., professional experience, therapeutic approach, patient group, type of therapy experience, and experience with online psychotherapy before the pandemic, as well as socio-demographic data, e.g., age and gender. The original questionnaire (OQ) also included questions about therapeutic work during the pandemic, e.g., information about the therapists’ attitudes to preventing COVID-19 infection during face-to-face sessions (beliefs about the appropriateness of prevention in the form of: masks, distance keeping, hand disinfection, room disinfection, therapist vaccination, patient vaccination, COVID-19 survey, therapist testing, patient testing). The complete questionnaire consisted of 19 items.

Moreover, we used standardized questionnaires, each of which we describe in more detail below.

#### 2.2.2. Attitudes toward psychological online interventions

The Attitudes toward psychological online interventions (APOI; 12; polish adaptation: 13) consists of 16 items that assess respondents’ acceptance of internet interventions along four dimensions: (1) Skepticism and perception of risks (SCE, the dimension referring to doubts about the therapists’ professionalism and the sense of possible negative consequences due to the lack of direct contact between patient and therapist; reversed scale); (2) confidence in effectiveness (CON, the dimension relating to the sense of trust in the effectiveness of working online); (3) technologization threat (TET, the dimension referring to uncertainty about the effectiveness of help through the use of technology; reversed scale), and (4) anonymity benefits (ABE, the dimension referring to the belief that anonymity of contact can promote confidentiality and freedom of expression). To calculate the overall attitude, the polarity of the subscale scores for SCE and TET is reversed so that all subscales contribute equally to the total score. Higher scores thus reflect a more favorable attitude toward internet interventions. The general attitude indicates the extent to which attitudes toward online psychological interventions are positive. The scale has demonstrated moderate internal reliability in its original study, with a Cronbach’s alpha of 0.77 (12). In our study the instructions and the test items were modified to refer to online psychological interventions as “online psychotherapy.”

#### 2.2.3. Fear of contracting COVID-19 scale

The Fear of Contracting COVID-19 Scale (FCS COVID-19) (14) is based on the Fear of Contracting Aids Scale (15) and asks respondents to indicate their level of fear and concern in relation to infection-relevant situations. A higher total score indicates great fear and concern about contracting COVID-19. The scale has demonstrated high internal reliability in its original study, with a Cronbach’s alpha of 0.91 (14).

#### 2.2.4. Pandemic fatigue scale

The Pandemic fatigue scale (PFS) (16) assesses pandemic fatigue, which is understood as a component of two separate but highly correlated factors: (1) Information fatigue (IF) and (2) behavioral fatigue (BF), both of which contribute to the overall experience of pandemic fatigue. The scale has demonstrated good internal reliability in its original study, with a Cronbach’s alpha score of 0.82/0.86 for the IF factor, 0.72/0.79 for the BF factor, and 0.83/0.88 for the overall PFS (16).

#### 2.2.5. Hospital anxiety and depression scale

The Hospital anxiety and depression scale (HADS) (17) is a self-report scale with 14 items. The HADS consists of two scales: (1) Anxiety (HADS–A) and (2) depression (HADS–D). The scale does not contain items on symptoms describing somatic aspects. The Cronbach’s alpha, based on a review of the validation literature (18), is 0.83 for the anxiety subscale and 0.82 for the depression subscale.

#### 2.2.6. Social support questionnaire

The F-SozU K-14 is a short version of the original Social Support Questionnaire (19; Polish adaptation: Juczyński, unpublished). This short version indicates the subjectively perceived social support, which is understood as the result of interactions between the individual and his/her environment, independent of the actual social support received. The scale has demonstrated very good internal reliability with a Cronbach’s alpha of 0.94 in the original study and the Polish version.

#### 2.2.7. The sense of efficiency test

The sense of efficiency test (SET) (20) focuses on the sense of self-efficacy as a variable of the individual’s personal resources, i.e., the characteristics of the individual that constitutes his/her belief in the effectiveness of the actions taken. The test consists of 17 items. The scale has demonstrated good internal reliability in its original study, with a Cronbach’s alpha of 0.87.

### 2.3. Statistical analysis

Statistical analyzes for this study were carried out using Statistica 13.0StatSoft and R. First, descriptive statistics were calculated to characterize the study group. Subsequently, a correlation analysis was performed using Spearman’s correlation coefficient for quantitative data and a point-biserial correlation coefficient for qualitative (nominal) data (coded as dichotomous). To determine which factor caused the greatest differences in attitudes toward online psychotherapy, linear regression models were calculated for each scale of the APOI test. The variables whose correlations with the APOI scales turned out to be statistically significant were used as predictors. A stepwise regression method was employed, removing the predictor with the highest value in each iteration. This was repeated until only significant variables (at 0.05 significance) remained. Numerical predictors were standardized to z-scores and binary variables were coded as dummy variables (0/1).

## 3. Results

### 3.1. Descriptive characteristics of participants

The study participants were 177 psychotherapists from four European countries: Poland (*n* = 48), Germany (*n* = 44), Sweden (*n* = 49), and Portugal (*n* = 36). The participants ranged in age from 23 to 80 years, and the mean age of the study group was 47.0 years (SD = 13.8 years). The psychotherapists participating in the study lived and worked mainly in large cities (almost 70% of participants). A large proportion of them worked in private practice (59%) or in a combination of private practice and public health service (36%), and most worked with adult or adolescent patients (aged 16–18 years). In the whole group, women made up 70% (men 28%, non-binary 2%), which is in line with the fact that this profession is more often practiced by women than by men (21, 22). The theoretical approaches most represented in the sample are: the integrative, the cognitive-behavioral and the psychodynamic and psychoanalytic approaches. Descriptives for the sample are given in Table 1.

**Table 1:** Sociodemographic characteristics of the psychotherapists and basic characteristics of psychotherapist practice.

Sociodemographic characteristics—therapist	
Psychotherapists	*N* (%)
	177 (100%)
Country of practice	*N* (%)
Sweden	49 (27.7%)
Poland	48 (27.1%)
Germany	44 (24.9%)
Portugal	36 (20.3%)
Gender	*N* (%)
Female	124 (70.1%)
Male	50 (28.2%)
Non-binary	3 (1.7%)
Age (years)	M (SD); MIN–MAX
	47.0 (13.8); 23–80
Experience (years)	M (SD); MIN–MAX
	13.5 (10.4); 1–50
Education	*N* (%)
Psychology	136 (76.8%)
Pedagogy	11 (6.2%)
Sociology	3 (1.7%)
Medical education	16 (9.0%)
Others	11 (6.2%)
School of psychotherapy	*N* (%)
During a comprehensive psychotherapy course (4–5 years of relevant education)	44 (24.9%)
Completed 4–5 year psychotherapy course	54 (30.5%)
Psychotherapist certificate	79 (44.6%)
Therapeutic approach	*N* (%)
Cognitive-behavioral	69 (39.0%)
Integrative	52 (29.4%)
Psychodynamic and psychoanalitical	28 (15.8%)
Systemic	11 (6.2%)
Other approach	10 (5.6%)
Existential and Gestalt	5 (2.8%)
Erickson therapy	2 (1.1%)
**Basic characteristics—psychotherapist practice**	
Practice location	*N* (%)
Big city (more than 100,000)	123 (69.5%)
Small town (1,000 to 100,000 residents)	51 (28.8%)
Village (less than 1,000 residents)	3 (1.7%)
Workplace	*N* (%)
Only private psychotherapeutic practice	104 (58.7%)
Only psychotherapist in the public health service	10 (5.6%)
Work in private practice and in public health service	63 (35.7%)
Patient age group	*N* (%)
Kids (0–15 years old)	28 (15.8%)
*Not working with kids*	149 (84.2%)
Adolescents (16–18 years old)	67 (37.9%)
*Not working with adolescents*	110 (62.1%)
Adults	170 (96.0%)
*Not working with adults*	7 (4.0%)
Hours per week online (hours)	M (SD); MIN–MAX
	5.4 (6.9); 0–40
Hours per week total work (hours)	M (SD); MIN–MAX
	19.0 (11.0); 4–50

#### 3.1.1. Dependent variables

In the present study, the dependent variable is the attitude toward online psychotherapy, measured by the overall score in the APOI (General Scale, M = 51.8, SD = 9.4, α = 0.85). We also analyzed attitudes toward online therapy in four detailed subscales: anonymity benefits (ABE, M = 10.1, SD = 2.7, α = 0.48); Confidence in Effectiveness (CON, M = 15.8, SD = 2.9, α = 0.82), Skepticism and Perception of Risks (SCE, M = 14.5, SD = 3.2, α = 0.79) and Technologization Threat (TET, M = 11.4, SD = 4.1, α = 0.85).

#### 3.1.2. Predictive variables

The selected predictors were divided into three variable clusters based on their apparent similarity, which are summarized in Table 2. Cluster 1 includes four dimensions related to the participants’ attitude toward COVID-19; Cluster 2 includes aspects of the therapists’ personal characteristics; and Cluster 3 includes variables related to various aspects of psychotherapeutic practice.

**Table 2:** Selected factors related to the shaping attitudes toward online psychotherapy among therapists—output model.

Cluster 1: attitudes toward COVID-19 pandemic	Cluster 2: therapist’s personal traits	Cluster 3: therapeutic practice
COVID-19 anxiety	Anxiety (HADS-A)	Therapeutic approach
COVID-19 prevention	Depression (HADS-D)	Client age group
Information fatigue	Sense of Efficacy (SET)	Therapy structure
Behavioral fatigue	Social Support (FSOU)	Professional experience
	Gender	Previous online experience
	Therapist age	

The results of the conducted research show that general fear of COVID-19 is not related to the confidence in the effectiveness of online therapy. However, when analyzing the correlations between a specific object of fear of COVID-19 and attitudes toward online therapy, some dependencies can be found here, as the results in Table 3 show. Thus, the Anonymity Benefits (ABE), Skepticism and Perception of Risk (SCE), Technologization Threat (TET), and general attitude toward online therapy (GS) are correlated with COVID-19 anxiety.

**Table 3:** Attitudes toward COVID-19 and attitudes toward online psychotherapy (only significant correlations).

	Attitudes to online therapy				
	Confidence in effectiveness (CON)	Anonymity benefits (ABE)	Skepticism and perception of risks (SCE)	Technologization threat (TET)	Attitudes to online psychotherapy (general scale)
**COVID-19 anxiety**					
Contacting COVID-19	0.03	**0.15** ^*^	0.01	0.09	0.09
Meeting people due to COVID-19	−0.09	**0.15** ^*^	−0.05	−0.01	−001
Having severe complication due to COVID-19	0.05	**0.15** ^*^	**0.15** ^*^	**0.20** ^**^	**0.19** ^*^
Dying from COVID-19	0.01	0.14	**0.17** ^*^	**0.20** ^**^	**0.18** ^*^
**COVID-19 prevention**					
Wearing masks during the session	**−0.18** ^*^	0.01	−0.03	−0.05	−0.09
Keeping distance during the session	0.10	**0.16** ^*^	**0.19** ^*^	0.13	**0.19** ^**^
Hand disinfection before the session	−0.01	**−0.17** ^*^	−0.11	−0.18	**−0.16** ^*^
Client vacintion	−0.10	**−0.16** ^*^	−0.05	−0.10	−0.14
COVID-19 survey	−0.05	**−0.17** ^*^	0.14	0.03	0.00
Client testing	−0.08	0.11	**0.18** ^*^	0.11	0.11
Therapist testing	−0.12	0.39	**0.21** ^*^	0.19	**0.21** ^*^
Pandemic fatigue					
Pandemic Information fatigue	−0.13	**−0.15** ^*^	**−0.18** ^*^	−0.13	**−0.2** ^**^
Pandemic behavior fatigue	−0.13	**−0.24** ^**^	**−0.27** ^***^	**−0.15** ^*^	**−0.26** ^***^

^*^Significant at 0.05 level; ^**^Significant at 0.01 level; ^***^Significant at 0.001 level.

The research results also show that the belief in the validity of certain preventive measures against COVID-19 transmission used by therapists in direct contact with patients during face-to-face sessions is related to therapists’ attitudes toward online therapy (see Table 3). Thus, confidence in wearing masks during face-to-face sessions is negatively correlated with confidence in the effectiveness of online therapy (CON, *r* = −0.18, *p* < 0.05). On the other hand, therapists who are convinced of the need to maintain physical distance with the patient during face-to-face sessions also appreciate the benefits of anonymity during online sessions (ABE; *r* = −0.16, *p* < 0.05), have a higher level of skepticism and risk perception associated with therapy sessions over the internet (TET, *r* = −0.19, *p* < 0.05), but they also have more positive attitudes toward online therapy in general (GS, *r* = −0.19, *p* < 0.01).

Pandemic information showed a significant negative correlation with the dimensions: anonymity benefits (ABE, *r* = 0.15, *p* < 0.05), Skepticism and Perception of Risks (SCE, *r* = 0.18, *p* < 0.05) and Attitudes toward Online Psychotherapy (GS, *r* = 0.20, *p* < 0.05). Behavioral Fatigue, on the other hand, correlated negatively with: anonymity benefits (ABE, *r* = −0.24, *p* < 0.05), Skepticism and Perception of Risks (SCE, *r* = −0.27, *p* < 0.001), Technologization Threat (TET, *r* = −0.15, *p* < 0.05) and Attitudes toward Online Psychotherapy (GS, *r* = − 0.26, *p* < 0.001). In other words, the less the therapists wanted to follow the pandemic reports in the media and the less they adhered to the restrictions, the lower their willingness to engage in online therapy (Table 3).

It turns out that of the variables analyzed from Cluster 2, most were not related to attitudes toward online therapy. The only statistically significant relationship was the negative correlation between the therapists’ age and confidence in effectiveness (CON, *r* = 0.22, *p* < 0.01). In other words, the older the therapist, the less confidence he/she had in the effectiveness of online therapy (Table 3). Whereas therapist’s anxiety, depression, sense of self-efficacy and social support received from others to the therapist and gender were insignificant.

The correlations between variables from Cluster 3, i.e., those related to therapeutic practice, and attitudes toward online therapy are presented in Table 4. As mentioned earlier, point-biserial correlations were presented for nominal (dichotomous) variables, and correlations for the quantitative variable were presented by Spearman’s correlations.

**Table 4:** Psychotherapeutic practice and therapists’ attitudes toward online psychotherapy (only significant correlations).

	Attitudes to online theraphy				
	Confidence in effectiveness (CON)	Anonymity benefits (ABE)	Skepticism and perception of risks (SCE)	Technologization threat (TET)	Attitudes to online psychotherapy (general scale)
**Therapeutic approach (point-biserial correlations)**					
Integrative	−0.06	−0.08	0.12	**0.17** ^*^	0.07
Other approach (including existential, gestalt, Erikson therapy)	0.04	0.08	0.14	**0.18** ^*^	**0.16** ^*^
**Client age group (point-biserial correlations)**					
Youth	−0.01	0.02	**0.22** ^**^	**0.20** ^**^	**0.15** ^*^
Adults	0.08	−0.04	**0.23** ^**^	**0.20** ^**^	**0.18** ^*^
**Therapy structure (point-biserial correlations)**					
Individual paychotherapy with kids	0.10	−0.02	**0.16** ^*^	0.13	0.14
Group psychotherapy: couples therapy	0.02	−0.01	0.17*	**0.18** ^*^	0.14
**Previous online experience (point-biserial correlations)**					
Experience in therapy online—before pandemic	0.07	0.11	**0.29** ^***^	**0.31** ^***^	**0.28** ^***^
Video session	0.04	0.05	**0.26** ^**^	**0.29** ^***^	**0.24** ^***^
Voice call	0.06	**0.19** ^*^	0.08	0.09	0.13

^*^Significant at 0.05 level; ^**^Significant at 0.01 level; ^***^Significant at 0.001 level.

Regarding the relationship between the therapeutic approach advocated by the therapist and his/her attitude toward online therapy, there was a statistically significant positive correlation between the integrative approach and Technologization Threat (TET, *r* = 0.17, *p* < 0.05).

We also noted some statistically significant differences in the relationship between therapists’ experience of working with different age groups of clients and attitudes toward online therapy. The dependencies are shown in Table 4. Furthermore, the structuring of therapeutic work and experience in online therapy before the pandemic correlate statistically significantly positively with attitudes to online therapy (Table 4).

#### 3.1.3. Merged regression model

In order to identify predictors of attitudes toward online therapy, five regression analyzes were performed, separately for the total score (general attitude) in the APOI questionnaire and for the four subscales of this questionnaire. For each model, potential predictors were selected based on the significant correlation of these variables with the dependent variable, as shown in the analyses above.

In the first regression model, predictors of Confidence in Effectiveness (CON) were looked for. Based on the correlations previously analyzed, the following predictors were included in the model: COVID-19 preventive behaviors – belief in wearing masks during face-to-face sessions (*β* = −0.14; *p* = 0.08) and therapist’s age (*β* = −0.13; *p* = 0.09). The model turned out to be statistically significant [*F*(2.174) = 4.52, *p* < 0.01, *R^2^* = 0.04], but confidence in effectiveness of online psychotherapy (CON) was not predicted by any of these predictors (Table 5).

**Table 5:** Statistically significant predictors of therapists’ attitudes toward online therapy.

Predictors (APOI)	β (95% confidence interval)	*p*-value
**Predictors of confidence in effectiveness (CON)**		
-	-	-
**Predictors of anonymity benefits (ABE)**		
COVID-19 prevention—keeping distance during the session	0.30 (0.15–0.44)	0.001 (*p* < 0.001)
Pandemic behavior fatigue	−0.27(−0.45–-0,10)	0.002 (*p* < 0.01)
Previous online therapy experience (voice call)	−0.18 (−0.32–-0.05)	0.009 (*p* < 0.01)
**Predictors of skepticism and perception of risk (SCE)**		
COVID-19 prevention—keeping distance during the session	0.17 (0.02–0.31)	0.02 (p < 0,05)
Pandemic Behavioral Fatigue	−0.26 (−0.42–-0.10)	0.001 (*p* < 0.001)
Experience in working with youth	-0,20 (−0.35–-0.05)	0.010 (*p* < 0.01)
Experience in working with adults	-0,19 (−0.34–-0.05)	0.008 (*p* < 0.01)
Previous online therapy expirience	0,21 (−0.13–0.29)	0.01 (*p* < 0.01)
**Predictors of technologization treat (TET)**		
Pandemic behavioral fatigue	−0.14 (−0.27–−0.001)	0.05 (*p* < 0.05)
Experience in working with youth	−0,18 (−0.32–−0.04)	0.01 (*p* < 0.01)
Experience in working with adults	−0.17 (−0.31–−0.04)	0.01 (*p* < 0.01)
Previous online therapy expirience	0.24 (0.15–0.33)	0.008 (*p* < 0.01)
**Predictors of attitudes to online therapy (general scale)**		
COVID-19 prevention—keeping distance during the session	0.24 (0.10–0.39)	0.001 (*p* < 0.001)
COVID-19 prevention—hand disinfection before the session	−0.16 (−0.30–−0.02)	0.02 (*p* < 0.01)
Pandemic behavioral fatigue	−0.21 (−0.37–−0.04)	0.01 (*p* < 0.01)
Experience in working with adults	−0.15 (−0.29–−0.01)	0.03 (*p* < 0.05)

The second regression model was constructed to search for predictors of Anonymity Benefits (ABE). The model turned out to be statistically significant [*F*(10.166) = 5.07, *p* < 0.001] and explained 19% of the outcome variance. Significant predictors of Anonymity Benefits (ABE) were: COVID-19 prevention—belief in keeping distance during face-to-face sessions, Pandemic Behavioral Fatigue, and previous online therapy experience (voice call). The results of the analysis are presented in Table 5.

The third regression model was constructed for the dependent variable—Skepticism and Perception of Risk (SCE). It proved to be statistically significant [*F*(13.163) = 6.09, *p* < 0.001] and explained 32% of the outcome variance. Among the predictors of Skepticism and Perception of Risk (SCE) analyzed, the following were found to be statistically significant: COVID-19 prevention – belief in keeping distance during face-to-face sessions, Pandemic Behavioral Fatigue, experience in working with adolescents, experience in working with adults, experience in conducting online therapy before the pandemic. Table 5 summarizes the results.

The fourth regression model for the dependent variable—Technologization Threat (TET) also turned out to be statistically significant [*F*(10.166) = 5.64, *p* < 0.001] and explained 25% of the outcome variance. Significant predictors of Technologization Threat (TET) were: COVID-19 prevention—belief in keeping distance during face-to-face sessions, Pandemic Behavioral Fatigue, experience in working with youth, experience in working with adults, experience in conducting online therapy before the pandemic. Detailed statistical indicators for this analysis are presented in Table 5.

**Figure 1:**
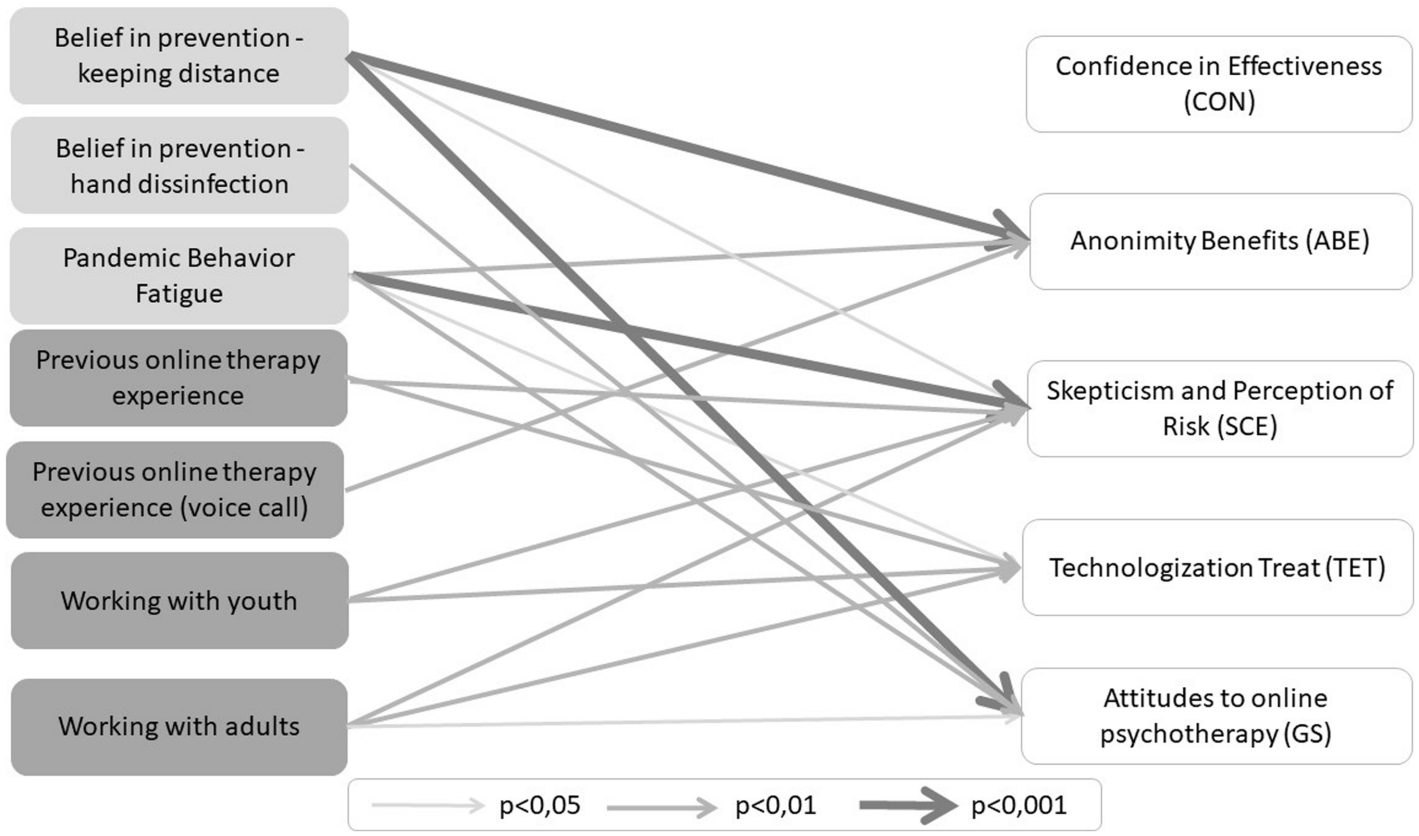
Predictors of therapists' attitudes toward online therapy.

Finally, the fifth regression model was carried out for the general attitude of the APOI questionnaire (general scale, GS), indicating the overall attitude of therapists toward online therapy. The constructed model explained 29% of the outcome variance and was statistically significant [*F*(12.164) = 5.45, *p* < 0.001]. Significant predictors of the general attitude of therapists toward online therapy turned out to be: COVID-19 prevention—belief in keeping distance during face-to-face sessions and hand disinfection before the session, Pandemic Behavioral Fatigue and experience working with adults. The results of the analysis are shown in Figure 1.

In summary, the strongest predictors were: (1) COVID-19 belief in prevention—keeping distance during face-to-face sessions for Anonymity of Benefits (ABE, *β* = 0.30) and for General Attitudes to Online Therapy (GS, *β* = 0.24); (2) Pandemic Behavioral Fatigue for Skepticism and Perception of Risk (SCE, *β* = 0.27); and (3) experience in conducting online therapy before the pandemic for Technologization Treat (TET, *β* = 0.24).

## 4. Discussion

Online psychotherapy is becoming increasingly popular. In this study, we aimed at investigating which factors (related to the COVID-19 pandemic itself, the psychotherapist, and the psychotherapeutic practice) influence therapists’ attitudes toward online psychotherapy.

Correlation analysis regarding COVID-19 pandemic attitudes (Cluster 1) showed that all selected predictors were associated with attitudes toward online therapy. Therapists who exhibited a higher level of fear of COVID-19 infection and health complications were more appreciative of the benefits of the anonymity of online therapy. The fear of health impairment and death was generally conducive to positive attitudes toward online therapy, regardless of some skepticism about the new way of working with patients. During the COVID-19 pandemic the real risk of infection led to a sense of respect for oneself and for loved ones (23). It is noteworthy that professionals who were in direct contact with COVID-19 patients exhibited more symptoms of depression, anxiety, insomnia, and stress in comparison with professionals who were not directly exposed to the virus (24). It is therefore not surprising that fear of infection is associated with more positive attitudes toward online therapy—also as a form of prevention.

The present study also shows that the belief in the validity of COVID-19 preventive measures used by therapists in direct contact with patients during face-to-face sessions is related to therapists’ attitudes toward online therapy. Interestingly, those therapists who were not convinced of the effectiveness of online therapy more often chose preventive measures during face-to-face sessions, such as covering their mouth and nose with a mask. It is worth highlighting that despite months of studies by experts from around the world, much is still unknown about the virus and prevention remains the most important strategy to control and contain the spread of the virus (25).

Our study showed that the fatigue experienced by therapists due to media reports about the pandemic and compliance with respective restrictions were associated with more negative attitudes toward online therapy. In fact, recent studies (16, 26, 27) have all pointed out that individuals experiencing higher levels of fatigue due to pandemic information are more likely to be demotivated to endure in protective behaviors and do not engage in seeking information about the pandemic. Therefore, being overloaded with pandemic information, some of which is fake or unverified, leads to fear, anxiety, restlessness, stress or feeling lost (28). Interestingly, information and behavioral fatigue were not statistically significantly associated with confidence in the effectiveness of online therapy. In their cross-sectional survey of psychotherapists’ attitudes toward online psychotherapy during COVID-19, Békés and Aafjes-van Doorn (2) indicate that while therapists from different parts of the world were generally successful in finding their way in online therapy, therapist fatigue and lack of confidence were related to negative attitudes to tele-psychotherapy, emphasizing the need for training, support, and supervision.

Analyzes of the therapists’ personal characteristics (Cluster 2) revealed that older therapists perceived online therapy as less effective. Studies on online psychotherapy conducted before the outbreak of the pandemic suggest an unclear relationship between age and attitudes toward online therapy (29, 30) and less frequent use of this form of work by older therapists (31). However, we suspect that older therapists had more time to refine their methods, clinical strategies, and techniques during face-to-face sessions. The need to change how psychotherapy is delivered may therefore be perceived as more threatening, reflecting their perceptions of online effectiveness.

Analyzes on therapeutic practice (Cluster 3) revealed that the therapeutic approaches, mainly the therapists following the integrative approach presented more technologization threat. There are several reports (32–35), although partly inconsistent, which indicate the relationship between the therapeutic approaches and the attitudes toward applying online interventions. A systematic review (36) conducted before the pandemic suggests that online psychotherapy showed similar effects compared with face-to-face therapies, however most studies involved cognitive behavioral therapy.

We have also observed that therapists working with adolescents and adults are generally more positive about online therapy, although they perceive some risk and technological threat associated with this form of therapy. Possibly, the awareness that these client age groups use media in a broad socio-educational and professional context may facilitate online therapy as an alternative therapy environment. A recent systematic review (37) examining the effectiveness of online mental health interventions for adolescents found online interventions to be effective in treating a variety of mental health conditions in adolescents, e.g., anxiety (38), depression (39), conduct disorders (40), or substance abuse (41). A review by Clarke et al. (42) indicates the significant positive effect of computerized cognitive behavioral therapy on anxiety and depression symptoms in adolescents and adults.

Regarding the structuring of therapeutic work, therapists providing individual therapy with children or couples were more skeptical and acknowledged the risks of online therapy. This may be due to the specific techniques and methods of therapeutic work with children which may be more difficult to apply in online therapy. Despite the studies indicating the general satisfaction with tele-therapy for children and adolescents (43, 44), the study conducted by Hopkins and Pedwel (45) during the 2020 lockdown found that the clinicians considered younger children as particularly disadvantaged by mental health services via tele-health, while the benefits were found primarily in adolescents. Meininger et al. (46) suggest that previous experience with video-delivered treatments, as well as the age of children and severity of symptoms, have an effect on the satisfaction with tele-therapy. Couples therapy also brings new challenges, such as using space and dealing with conflict escalation remotely. The study by Machluf et al. (47) demonstrates that although therapists tend to view couples therapy positively, they have major concerns about establishing a strong therapeutic bond with both partners, dealing with escalating conflicts and the risk of treatment dropout.

We also found that therapists who had previous experience with online interventions prior to the COVID-19 pandemic exhibited more positive overall attitudes toward psychological online interventions. This finding is in line with the results of previous studies (2, 48). Nevertheless, therapists who had experience with online sessions before the pandemic were also skeptical, recognizing the risk of interventions via the internet and technological threat. Lack of face-to-face contact, network failure, delayed video or audio transmission and other technological difficulties affect the communication process in online therapy and are consequently associated with feelings of frustration and anxiety on the part of both therapist and client (49).

The identification of predictors of attitudes toward online interventions showed that none of the selected predictors were predictive of confidence in the effectiveness of online psychotherapy. In turn, COVID-19 prevention – belief in keeping distance during face-to-face sessions, pandemic behavioral fatigue, and previous online therapy experience (voice call) were again predictors of the benefit from anonymity of online therapy.

Undoubtedly, the increase in online consultations has enabled patients to participate anonymously in internet interventions wherever and whenever they want, which results in reducing the barrier to treatment and the use of new forms of therapy (50). However, one of the basic factors influencing the therapists’ sense of control is whether clients can disclose the necessary information during sessions rather than remaining anonymous (51). If such information is missing and the therapist feels that he/she is losing control, the whole online counseling experience is likely to change (52). Tsalavouta (53) found that the therapists’ experience of lack of control, in terms of managing risk-related situations, was perceived to be anxiety-provoking, whereas online therapists feel reassured when they are able to control or manage risk situations (54).

Maintaining preventive behaviors such as keeping distance during face-to-face sessions, pandemic behavioral fatigue, previous online therapy experience as well as experience in working with youth and adults are predictors of skepticism and risk perception. There are studies (34, 55) pointing out that therapists are reserved and careful about online therapy and that those who have previous experience with online therapy are more likely to display skepticism and more adequately assess risk. The literature review (56–59) shows that therapists working online face numerous issues, such as establishing strong therapeutic alliance online, difficulties in expressing emotions or perceiving non-verbal behaviors, the risk of technical shortcomings, insufficient internet skills, and weak personal belief in the effectiveness of online therapy. In addition, pandemic behavioral fatigue can lead to mental weariness and loss of motivation, therefore reducing the focus on identifying possible risks of online therapy.

Experience with online therapy prior to the pandemic, pandemic behavioral fatigue, and experiences with adolescents and adults were predictors of technologization threat. To date, online therapy has been described as a solution for expatriates or people living in rural areas, as well as for people with mobility problems or in poor health (60, 61). In contrast, the pandemic has shifted the benefit–risk balance in favor of tele-health and online work, indicating that patient characteristics and therapist characteristics are most important for the effectiveness of the treatment effectiveness whether it is remote or face-to-face (62–64). Technological problems during therapeutic sessions are associated with feelings of frustration and anxiety (49).

The present study demonstrated that the use of preventive measures, such as hand sanitization before the session, pandemic behavioral fatigue, and experience in working with adults were significant predictors of therapists’ negative attitudes toward online psychotherapy. In contrast, belief in prevention in the form of keeping distance during face-to-face session was found to have a positive effect on overall attitudes toward online therapy. Our findings therefore suggest that psychotherapists’ attitudes toward online psychotherapy are influenced not only by factors related to the therapeutic process (therapeutic approach or clinical experience) but also by pandemic behavioral fatigue, namely following news reports and adhering to imposed restrictions. This is consistent with previous findings by Békés and Aafjes-van Doorn (2), who showed that therapists’ fatigue and lack of confidence were associated with negative attitudes to tele-psychotherapy.

Our study has several limitations. The study included psychotherapists from four European countries, so the results cannot be plainly generalized to psychotherapists residing in other countries. Furthermore, despite our best efforts, this is a convenience sample which cannot reflect the full diversity of psychotherapists’ responses. In particular, recruitment of therapists via internet platforms could have led to selection bias by including mainly therapists, who are generally open to modern technologies. In addition, our study was based only on self-report, which we know can lead to social desirability and bias the results. However, due to the anonymity of the survey method, this should not have a great impact. Notwithstanding these limitations, this study also sheds light on future studies. Particularly, it may be interesting to investigate whether clients’ perceptions of the effectiveness of online therapy are similar to those of therapists and to include objective measures of clients’ treatment outcomes.

In conclusion, this international research showed that the predictors that were significant for psychotherapists’ attitudes toward online therapy during the pandemic were belief in prevention – keeping distance and hand sanitization, pandemic behavioral fatigue, previous online therapy experiences, in particular in the form of voice calls, as well as working with adolescents and adults alike. This highlights the need to ensure the comfort and quality of the therapists’ work and also draws attention to the importance of proper training in providing online therapy to dispel negative beliefs about online therapy.

## Data availability statement

The datasets presented in this article are not readily available because the study was conducted by working psychotherapists and not making the data set publicly available is related to ethical considerations and preventing a situation in which any of the study participants could be identified. Requests to access the datasets should be directed to emiliapsycholog@gmail.com.

## Ethics statement

The studies involving human participants were reviewed and approved by Ethical Committee of the Institute of Psychology of the University of Szczecin, nr KB 18/2021. The patients/participants provided their written informed consent to participate in this study.

## Author contributions

ER and JF contributed to the conception and design of the study. ER, JF, HL, CM, MM, and JM organized the database. HL and ER performed the statistical analysis. ER, JF, HL, CM, MM, NS, JM, VT, AA, and DB wrote the first draft of the manuscript and wrote sections of the manuscript. All authors contributed to the article and approved the submitted version.

## Conflict of interest

The authors declare that the research was conducted in the absence of any commercial or financial relationships that could be construed as a potential conflict of interest.

## Publisher’s note

All claims expressed in this article are solely those of the authors and do not necessarily represent those of their affiliated organizations, or those of the publisher, the editors and the reviewers. Any product that may be evaluated in this article, or claim that may be made by its manufacturer, is not guaranteed or endorsed by the publisher.
